# Technological Interventions and Social Participation in Community-Dwelling Older Adults With or Without Dementia

**DOI:** 10.1093/geroni/igab046.2255

**Published:** 2021-12-17

**Authors:** Pascale Heins, Lizzy Boots, Wei Qi Koh, An Neven, Frans Verhey, Marjolein de Vugt

**Affiliations:** 1 Maastricht University, Maastricht, Limburg, Netherlands; 2 Alzheimer Centrum Limburg, Maastricht University, Maastricht, Limburg, Netherlands; 3 National University of Ireland Galway, Galway, Galway, Ireland; 4 UHasselt - Hasselt University, Transportation Research Institute (IMOB), Diepenbeek, Limburg, Belgium

## Abstract

Social isolation is a growing health issue in community-dwelling older adults with and without dementia as it can negatively affect their health and well-being. Consequently, psychosocial interventions targeting their social participation are increasingly gaining importance. So far, however, little is known about the potential of technological interventions in this population. Therefore, this systematic review explored the effectiveness of technological interventions in improving social participation of community-dwelling older adults with and without dementia. Records identified through five scientific databases were independently screened by two reviewers. A total of 36 studies published between 2005 and 2020 were included in a narrative synthesis. Studies differed widely in study design, type of technology, used outcome measures, and methodological quality. However, the findings highlight the potential role of technological interventions in improving different dimensions of social participation. At the same time, barriers and facilitators of these interventions to social participation were identified.

